# Onyx Embolization of Ruptured Intracranial Aneurysm Associated with Behçet's Disease

**DOI:** 10.1155/2013/797045

**Published:** 2013-09-24

**Authors:** Maher Kurdi, Saleh Baeesa, Mohammed Bin-Mahfoodh, Khalil Kurdi

**Affiliations:** ^1^Pathology Department, Faculty of Medicine, King Abdulaziz University, Jeddah 21589, Saudi Arabia; ^2^Division of Neurological Surgery, Faculty of Medicine, King Abdulaziz University, Jeddah 21589, Saudi Arabia; ^3^Neurosciences Department, King Faisal Specialist Hospital and Research Center, Jeddah 21499, Saudi Arabia; ^4^Radiology Department, King Faisal Specialist Hospital and Research Center, Jeddah 21499, Saudi Arabia

## Abstract

*Introduction*. Intracranial aneurysms associated with Behçet's disease (BD) are a rare occurrence. They are fragile, thin-walled pseudoaneurysms, which have high tendency to rupture and present a therapeutic challenge. *Case Presentation*. We report a 26-year-old male with BD presented with subarachnoid hemorrhage due to ruptured middle cerebral artery aneurysm. Additionally, two unruptured aneurysms were identified. He underwent endovascular embolization using Onyx with successful obliteration of the ruptured aneurysm. Medical therapy resulted in regression of one and resolution of the other aneurysms. *Conclusion*. We describe the first report of the application of Onyx for obliteration of ruptured cerebral aneurysm in BD as a feasible and safe therapeutic option for patients who are not candidates for other techniques.

## 1. Introduction

Behçet's disease (BD) is a multisystem inflammatory disorder of unknown etiology characterized by recurrent oral and genital ulcerations, optic manifestations of uveitis, iritis, and retinitis, and dermatological manifestations of erythema nodosum and pseudofolliculitis [1]. The most important morbidity encountered during the disease progression is vascular involvement with overall incidence of 7–29% [2]. Although relatively common in systemic visceral and peripheral vessels, the involvement of intracranial vessels is extremely rare, with overall incidence of 0.3–1.5% [3–5]. Because of the high risk of mortality from ruptured intracranial aneurysms in BD, a high suspicious index and diagnostic imaging surveillance are recommended. Symptomatic intracranial aneurysms are challenging to treat due to their configuration and distal location, and they require either urgent surgical clipping or endovascular treatment.

We describe a patient with BD who experienced a subarachnoid hemorrhage (SAH) secondary to a ruptured right fusiform middle cerebral artery (MCA) aneurysm associated with multiple aneurysms of the anterior and posterior circulations. He was treated by endovascular embolization of the ruptured aneurysm, for the first time in the literature to our knowledge, using liquid embolic agent Onyx (an ethylene vinyl alcohol copolymer) with successful preservation of the parent artery; medical therapy followed for the asymptomatic smaller aneurysm.

## 2. Case Report

A 26-year-old Saudi Arabian male, with a 9-month diagnosis of BD, presented on October 2010 with sudden onset of headache and generalized tonic clonic seizure. His initial computed tomography (CT) scan of the brain report revealed a significant SAH in the right sylvian fissure. He was then transferred to our institution for definitive management.

**Figure 1:**
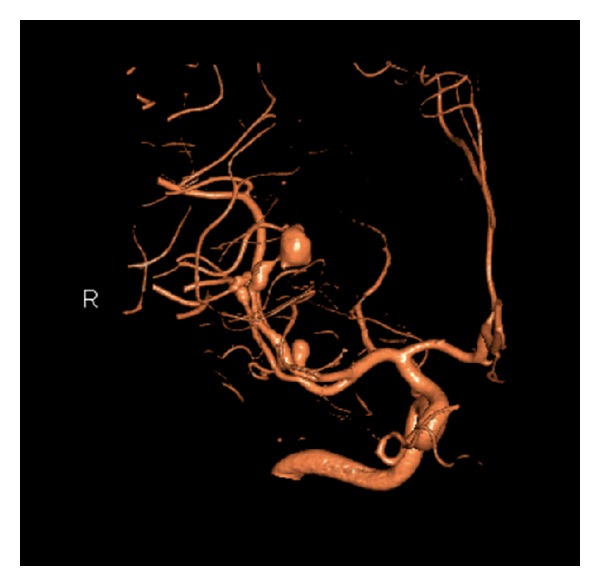
Oblique CTA scans demonstrating an ectatic fusiform dilatation of the involved MCA distal branch with aneurysm, in addition to saccular smaller aneurysm at the origin of MCA bifurcation.

**Figure 2:**
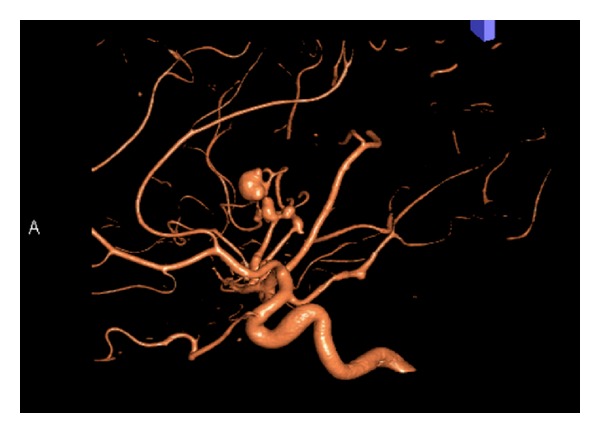
Lateral CTA scans demonstrating an ectatic fusiform dilatation of the involved MCA distal branch with aneurysm. In addition, a saccular smaller aneurysm at the origin of MCA bifurcation and another fusiform smaller aneurysm at the first segment of PCA were demonstrated.

**Figure 3:**
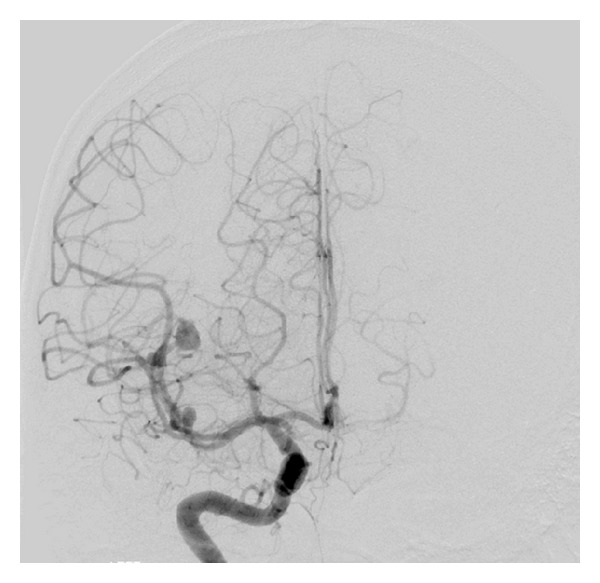
Anterior-posterior view of cerebral angiographic (DSA) image demonstrating an 8 mm right M3-MCA fusiform aneurysm and a 4 mm M2-MCA saccular aneurysm.

**Figure 4:**
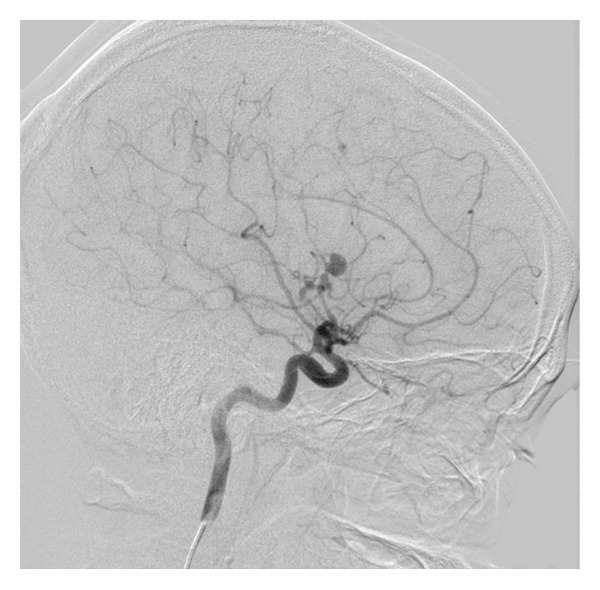
Lateral view of cerebral angiographic (DSA) image demonstrating an 8 mm right M3-MCA fusiform aneurysm with disease ectatic parent artery.

General physical examination revealed normal vital signs, with mild neck rigidity. He had a mild sclera injection, and there were with no skin or mucosal lesions. The neurological examination revealed an alert, oriented patient without focal neurological deficit. Routine blood investigations, including complete blood count, electrolytes, and renal and coagulation profiles, were within normal values. A subsequent brain CT scan, on the 5th SAH day, was negative for SAH but 3D-CT angiogram revealed one large distal fusiform and another 2 smaller aneurysms: one at the right MCA and another at the right PCA (Figures 1 and 2). A conventional four-vessel cerebral angiogram (DSA) revealed an ectatic fusiform dilatation of insular M3-segment of the right MCA with 4 mm proximal (M2-segment) aneurysm and 8 mm distal (M3-segment) fusiform aneurysms (Figures 3 and 4). The left carotid and posterior circulations were normal, apart from a 3 mm proximal PCA aneurysm.

The patient was deemed ineligible for conventional therapy due to the small size of the parent artery which makes its preservation and protection not possible and carries a higher risk of rerupture and bleeding. The procedure was discussed with the patient and his family and an informed consent was obtained. Endovascular embolization using Onyx within the ruptured aneurysm was considered as a viable treatment option.

**Figure 5:**
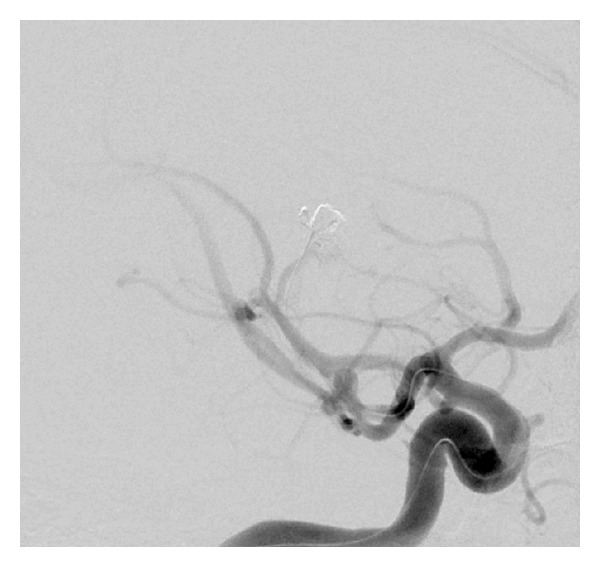
Intraprocedure cerebral angiogram demonstrating microcatheter tip placement within the fusiform aneurysm and Onyx embolization completion.

Under general anesthesia, a cerebral angiogram was performed through a right femoral artery puncture. A size 6 French guiding catheter (Guider Soft-tip, Boston Scientific) was placed in the right internal carotid artery. A Marathon microcatheter (1.5F/1.7F eV3 Neurovascular) was advanced over a silver speed 10 microwire and placed in the ruptured aneurysm against the wall. Onyx 18 was injected slowly into the aneurysm with slow lamination of the precipitated amount within the aneurysm (Figure 5). Control of Onyx 18, to avoid any penetration of the normal distal artery, was assured to allow retrograde filling of the distal normal artery via collaterals. Complete occlusion of the ruptured aneurysm was successful and filling with Onyx 18 achieved. This also resulted in cessation of flow within the fusiform dilated parent artery and the associated proximal aneurysm. Immediate retrograde filling of normal branch distal to the aneurysm was observed on the late arterial phase. A postprocedural cerebral angiogram confirmed the complete obliteration of the diseased segment in the right MCA, including the ruptured fusiform aneurysm, and the smaller aneurysm was left for medical therapy.

**Figure 6:**
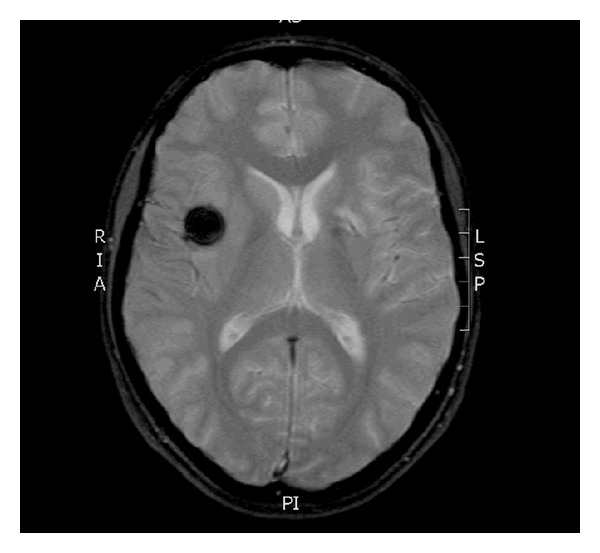
MRI-GE image scan 24 hours after Onyx embolization showing an area of low intensity at the site of embolic material within the aneurysm without ischemic complication.

**Figure 7:**
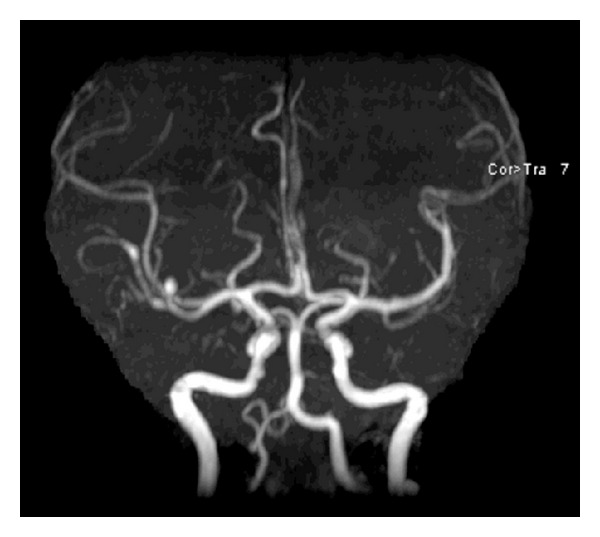
24-hour post-Onyx embolization MRA scan in AP view demonstrating resolution of the embolized MCA aneurysm.

Postprocedure period was uneventful; the patient was extubated and had no neurological deficits. There was no evidence of postendovascular complications on follow-up magnetic resonance imaging (MRI) and MR angiogram (MRA) scans in the next day. An area of low intensity at the site of embolic material has been revealed with resolution of the ruptured MCA aneurysm (Figure 6). Furthermore, there was no evidence of any ischemic insult (Figure 7). The patient was started on a 6-month course of prednisolone (1 mg/kg/day), azathioprine (150 mg/day), and colchicine (1 mg/day) for treatment of the remaining small aneurysm.

**Figure 8:**
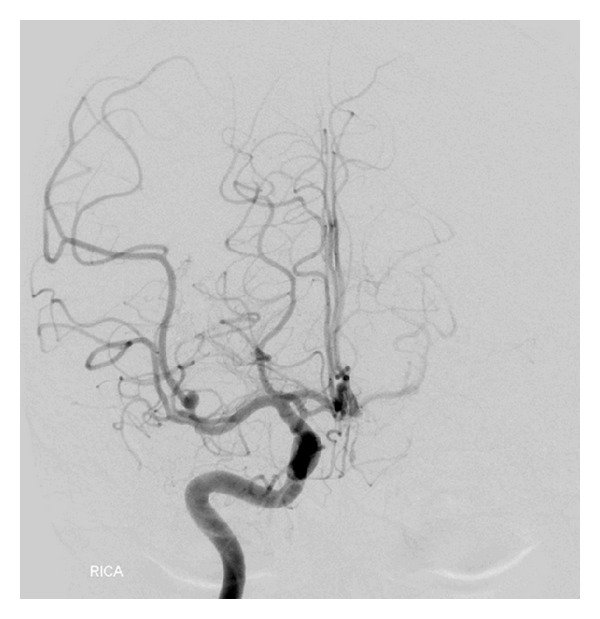
Cerebral angiographic (DSA) image in AP view of right ICA at 6-month follow-up, demonstrating complete obliteration of the embolized aneurysm with retrograde filling of the normal artery distal to the aneurysm. The smaller aneurysm has shown significant reduction in size.

**Figure 9:**
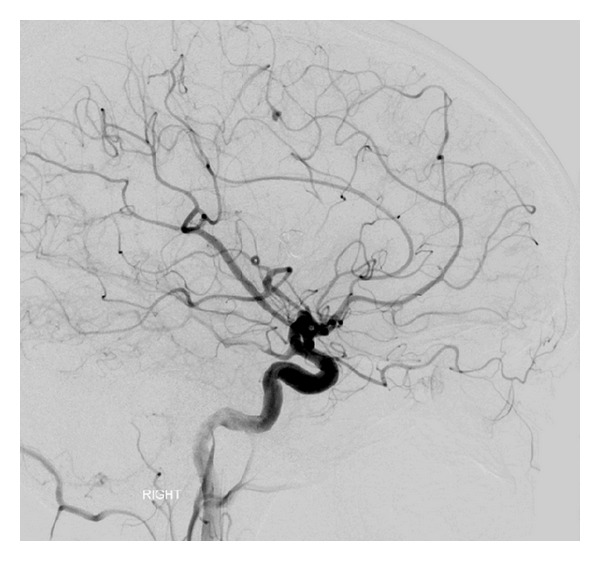
Cerebral angiographic (DSA) image in lateral view of right ICA at 6-month follow-up, demonstrating complete obliteration of the embolized aneurysm with retrograde filling of the normal artery distal to the aneurysm.

At 6 months of follow-up, DSA showed persistent occlusion of the right MCA fusiform aneurysm with 40% decrease in the size of the proximal MCA aneurysm, and resolution of proximal PCA aneurysm (Figures 8 and 9).

## 3. Discussion

The pathophysiology of vascular involvement in BD is believed to be an inflammatory related process where lymphocytic infiltrations of the vasa vasora occur leading to vasculitis and thickening of the vascular wall with subsequent thrombosis and occlusion. Less commonly, thinning of the tunica media and rupture of the internal and external membranes leading to weakness of the vessel wall and subsequent ectasia and aneurysm formation [1, 2, 5].

The overall incidence of vascular involvement in BD is 7–29% and much more frequently in men [1, 2]. Venous involvement in the form of venous thrombosis is more common (up to 85%) than arterial involvement, although both can coexist; arterial involvement, usually aortic and pulmonary vessels, occurs in 7% of patients with BD and consists of occlusion, aneurysm, or peudoaneurysm [2, 5]. Intracranial arterial aneurysm, usually of a peripheral location, is extremely rare in patients with BD. Three studies, two Moroccans and one Saudi Arabian, reported one case in each series with an incidence of 0.3–1.5% [3–5].

We reviewed the literature through PubMed search engine searching for intracranial aneurysms/SAH in BD, similar to our case. The search revealed description of reported cases for intracranial aneurysms in 22 patients with BD, in addition to our case, which were summarized in Table 1 [6–27]. Few reports mentioning the occurrence of aneurysms without patient data were not added to the table. Eighteen patient (78%) were males with mean age of 40 years (range, 12–65 years). The majority (87%) presented with ruptured aneurysm causing SAH, one with MCA thrombosis leading to hemispheric infarction, one with vertebral dissecting aneurysm causing lateral medullary syndrome, and one was an incidental finding. The ethnic distribution revealed that the majorities are from far eastern (Japan) and Mediterranean (Turkey) regions. Thirty-five aneurysms were identified on angiograms in these cases, 25 (71%) in the anterior circulation and 13 of them involving MCA as the commonest artery involved. Multiple aneurysms occurred in 7 patients (30%), which predominately involved the anterior circulation, particularly MCA. Our case suffered from SAH and presented with 2 saccular and fusiform aneurysms located in the MCA, which is extremely rare and the only case reported.

Treatment of the reported cases of ruptured cerebral aneurysms in BD varied from only medical treatment to microsurgical clipping or endovascular treatment (Table 1). Microsurgical treatment was performed in 10 patients; 8 had clipping of the aneurysms and 2 had excision, with postexcision grafting performed in one. Endovascular treatment was performed in 8 patients. Coiling technique was utilized in 5 patients; one patient had VA stent, and another one had NBCA embolization, and Onyx embolization was used in our patient. Endovascular treatment has recently been the first choice of therapy despite the concern that insertion of the catheter may induce formation of pseudoaneurysm at the puncture site, or thrombosis of the vessels. Two patients with ruptured aneurysm were treated only medically with immunosuppressive drugs (corticosteroids plus azathioprine or cyclophosphamide and colchicine). One patient received no therapy after spontaneous thrombosis of the VA aneurysm, and 2 died before they receive any treatment.

There was no consistency about the indication for adjuvant immunosuppressive therapy after securing the aneurysm. Five patients did not receive medical therapy after clipping or coiling of the aneurysm or after spontaneous resolution of the aneurysm [6, 15, 21, 22, 26]. Three patients, 2 with endovascular coiling and one with excision, did not receive adjuvant medical therapy [7, 9, 16]. Some authors emphasized the importance of receiving immunosuppressive drugs for few months, after clipping or coiling of the aneurysm, to prevent new aneurysm formation [11, 13, 14, 18–20, 23, 24]. Nevertheless, the use of immunosuppressive therapy for treatment of unruptured aneurysms may not be successful [10], which emphasizes the regular surveillance with noninvasive methods, such as MRI and MRA or CTA with high sensitivity instead of invasive procedure (DSA) [22].

Outcome could not be extracted in detail from all the reports but there was a mortality of 6 patients (26%). One patient gradually worsened and died due to the progression of the disease, and 3 patients died of severe subarachnoid bleeding and intracranial clot; the remaining patients were reported to be alive after treatment. Prognosis of ruptured intracranial aneurysms in patients with BD is unclear but is likely to be influenced by both the severity of the hemorrhage and the course of the disease [25]. On 2-year follow-up of our patient, he remained in a good condition and there were persistent occlusion of the Onyx embolized aneurysm and resolution of the smaller aneurysm after medical therapy.

The choice of treatment modality in our case was based on the presence of a small sized and diseased parent artery where preservation and protection were not possible; therefore, we did not consider surgical clipping. Endovascular treatment using coils would be technically difficult for the distal locating aneurysm, not protecting the parent artery, and carry a higher risk of rupture. Therefore, we decided to use Onyx 18 as a reasonable alternative therapeutic modality.

Onyx is a liquid embolic material, a polymer mixture of EVOH (ethylene vinyl alcohol copolymer) and DMSO (diethyl sulfoxide, opacified in micronized tantalum powder) which has a different concentration; Onyx 18 contains 6% copolymer and 94% DMSO. When onyx comes in contact with blood or water, the EVOH precipitates because of the rapid diffusion of DMSO solvent. Therefore, Onyx would precipitate and solidify into a spongy material capable of achieving permanent vascular occlusion. In contrast to other embolic materials (e.g., NBCA), Onyx has nonadhesive quality and longer working time, which minimize gluing to microcatheters or fracture and migration of embolized materials. It also can be injected several times without removing the catheter. Onyx embolization is used initially in the treatment of aneurysms associated with an AVM in the form of an intranidal ruptured aneurysm or postnidal ruptured venous varix. Its unique features for treating distally located and vasculitic thin-walled ruptured aneurysms have been demonstrated first by Utoh and colleagues (1995) using Onyx 18 to embolize a ruptured infectious PCA aneurysm [27]. With the recent advances in endovascular technology, flow diverters (pipeline embolization device) and high concentration Onyx (Onyx HD 500) have been used for treatment of large intracranial aneurysms with equally effective results [28]. Recently, cerebral aneurysm multicenter European Onyx trial, the first multicenter prospective study using Onyx embolization for cerebral aneurysms, demonstrated decreased risk for recanalization and complications of smaller aneurysms compared with coil embolization [29].

## 4. Conclusion

A high index of suspicion should be raised for the incidence of cerebral aneurysms in patients with BD. Medical treatment including corticosteroid and immunosuppressive therapies should be the initial part of the management of asymptomatic aneurysms with regular imaging surveillance. However, if they rupture, endovascular treatment using Onyx should be considered as a curative therapy among other treatment modalities.

## Figures and Tables

**Table 1:** Summary of reported cases in the literature of ruptured intracranial aneurysms associated with Behçet's disease.

	Authors (Year)	Age (yrs.)/sex	Presentation	Ethnicity	Location	Intervention	Additional
1	Katoh et al. (1985)	29/M	SAH	Japanese	MCA	Clipping	NS
2	Buge et al. (1987)	43/M	Cerebral infarction	Moroccan	ACA, ICA, MCA. PComA	No	Medical therapy
3	Kerr et al. (1989)	12/M	SAH	Caucasian	AComA, PComA, AChor	Clipping	Medical therapy
4	Tsuji et al. (1990)	62/F	SAH	Japanese	Bilateral MCA, ICA	Clipping	NS
5	Bahar et al. (1993)	40/M	SAH	Chinese	VA	Stent	Medical therapy
6	Khodja et al. (1991)	43/M	NS	Tunisia	AComA	No	Medical therapy
7	Dietl et al. (1994)	47/F	SAH/ICH	Turkish	Bilateral ICA	Coiling	Medical therapy
8	Ildan et al. (1996)	28/M	SAH	Turkish	AComA	Clipping	Medical therapy
9	Itoh et al. (1996)	65/M	Medullary infarction	Japanese	VA	No	No
10	El Abbadi et al. (1999)	44/M	SAH	Moroccan	Bilateral MCA	Clipping	NS
11	Nakasu et al. (2001)	57/M	SAH	Japanese	Bilateral MCA	Clipping	Medical therapy
12	Rosensting et al. (2001)	36/M	SAH	Armenian	SCA	Coiling	Medical therapy
13	Kizilkilic et al. (2003)	38/M	SAH	Turkish	SCA	Coiling	Medical therapy
14		55/M	SAH		VA	NBCA embolization	
15	Koçak et al. (2004)	37/M	SAH	Turkish	MCA	Clipping	Medical therapy
16	Zsigmond et al. (2005)	38/M	SAH	Mediterranean	AComA	Clipping	No
17	Chi and Deruytter (2005)	30/F	SAH	Japanese	SCA	Excision	No
18	Agrawal et al. (2007)	36/F	SAH	Indian	ICA	Coiling	Medical therapy
19	Kaku et al. (2007)	19/F	SAH	Japanese	Bilateral MCA	Excision and grafting	Medical therapy
20	Aktas et al. (2008)	38/M	SAH	Turkish	BA	No	No, patient died
21	Ozveren et al. (2009)	38/M	Unruptured	Japanese	ICA	Coiling	No
22	Senel et al. (2010)	45/M	SAH	Turkish	PCA	No, spontaneous thrombosis	No
23	Present Case (2010)	26/M	SAH	Saudi Arabian	Multiple MCA	Onyx embolization	Medical therapy

NS: not specified, MCA: middle cerebral artery, ICA: internal carotid artery, PComA: posterior communicating artery, PCA: posterior cerebral artery, AChorA: anterior choroidal artery, AComA: anterior communicating artery, VA: vertebral artery, BA: basilar artery, SCA: superior cerebellar artery, NBCA: N-butyl cyanoacrylate, SAH: subarachnoid hemorrhage, ICH: intracerebral hemorrhage.

## Abbreviations

NS: Not specified
MCA: Middle cerebral artery
ICA: Internal carotid artery
PComA: Posterior communicating artery
PCA: Posterior cerebral artery
AChorA: Anterior choroidal artery
AComA: Anterior communicating artery
VA: Vertebral artery
BA: Basilar artery
SCA: Superior cerebellar artery
NBCA: N-Butyl cyanoacrylate
SAH: Subarachnoid hemorrhage
ICH: Intracerebral hemorrhage.
